# Tracking the *Mandorla di Avola* Almond Variety by Means of ICP Analysis

**DOI:** 10.3390/foods13162634

**Published:** 2024-08-22

**Authors:** Federica Gulino, Cassandra Siragusa, Elisa Calà, Francesca Gullo, Maurizio Aceto

**Affiliations:** 1 Dipartimento per lo Sviluppo Sostenibile e la Transizione Ecologica (DISSTE), Università degli Studi del Piemonte Orientale, Piazza Sant’Eusebio, 5-13100 Vercelli, Italy; federica.gulino@uniupo.it (F.G.); cassandrasiragusa94@gmail.com (C.S.); elisa.cala@uniupo.it (E.C.); 2 Dipartimento di Scienza Applicata e Tecnologia (DISAT), Politecnico di Torino, Corso Duca degli Abruzzi, 24-10129 Torino, Italy; francesca.gullo@polito.it

**Keywords:** authentication, lanthanides, almond, ICP-MS, ICP-OES, traceability, chain, trace elements, Sicily

## Abstract

The *Mandorla di Avola* is recognized all over the world as one of the best almond varieties. It is cultivated in a small area inside the provinces of Siracusa and Ragusa (Sicily, southern Italy). It is used in traditional Sicilian cuisine for both salty and sweet foods and of course in artisan pastry, apart from being consumed as a fruit. Due to its extraordinary organoleptic and beneficial features, the *Mandorla di Avola* is frequently counterfeit with almond varieties of lower quality coming from other countries. While its nutraceutical features have been studied, the possibility of authenticating it with respect to other varieties has not been explored. In this work, we used microelements determined with ICP-OES and ICP-MS as chemical descriptors to distinguish samples of *Mandorla di Avola* almonds from almonds coming from California and Spain, which are usually employed as substitutes in pastry. Among the different elements determined, Mn and P were found to be the best descriptors for authentication.

## 1. Introduction

Almonds, the seed of the *Prunus dulcis* tree, are a highly appreciated fruit for its sensory and healthy features [1,2,3]. They have several uses in cooking, with a particular relevance to the sweets sector. Moreover, their by-products have relevant properties for sustainable food systems [4,5].

Among the different varieties cultivated, the *Mandorla di Avola* is recognized all over the world as one of the best. It is cultivated in a small area inside the provinces of Siracusa and Ragusa (Sicily, southern Italy) [6]. The famous Sicilian writer Leonardo Sciascia used to describe it as “the almond with a perfect oval”. The denomination *Mandorla di Avola* accounts for three different cultivars: *Pizzuta*, *Fascionello*, and *Romana* or *Corrente d’Avola*. These cultivars are used in traditional Sicilian cuisine for both salty and sweet foods and of course in artisan pastry, apart from being consumed as raw fruits. A typical, well appreciated, intermediate product of the *Mandorla di Avola* is the almond paste, obtained by milling almonds under a vacuum up to a size of 20 µm and used for the preparation of sweets or drinks.

The nutraceutical features of the *Mandorla di Avola* have been recently studied with respect to seeds and skins [7,8], husks [9,10], and leaves [11]. These studies highlighted the great polyphenol content and antioxidant activity of this variety, with particular relevance to its high content of α-tocopherol [12]. Also, its by-products deserved attention: Ingegneri et al. [13] studied the possible beneficial effects of blanched skin and blanched water obtained from these almonds.

However, the possibility of authenticating it with respect to other varieties has not been fully explored, which is an important issue if we consider that, due to its extraordinary organoleptic and beneficial features, it is frequently counterfeit with almond varieties of lower quality or coming from other countries [14].

The possibility of using physico-chemical markers to determine the origin and cultivar of almonds has been recently reviewed by Sanahuja et al. [15]. These authors recommended an accurate selection of variables, employment of metabolomics or DNA fingerprinting, and application of multivariate statistical techniques. More specifically, Barreira et al. [16] used organic markers and pattern recognition methods to discriminate between PDO (Protected Designation of Origin) Portuguese cultivars and commercial non-PDO cultivars. In a similar way, Kalogiouri et al. [17] exploited the profiles of phenolic compounds and tocopherols to discriminate between Texas almonds produced in USA and Greece. A spectroscopic approach was instead used by Arndt et al. [18] who used near-infrared (NIR) spectroscopy coupled with classification using a Support Vector Machine (SVM) model to discriminate between American and Mediterranean (Spanish + Italian) almonds. Trace element determination by means of ICP-MS has been used by von Wuthenau et al. [19,20] to discriminate among almonds of thirty different varieties, six different countries, and four harvest years; the authors found that harvest year and variety were less important than geographic origin. Amorello et al. [21] used trace elements and fatty acids as chemical markers to discriminate between almonds from Sicily, Spain, and California. The rate of correct classification was improved by combining both classes of markers.

As for the *Mandorla di Avola*, to the authors’ knowledge, only a few authentication studies have been conducted up to now, devoted specifically to this variety. Firmani et al. [22] developed a method based on Near Infrared (NIR) spectroscopy coupled with supervised pattern recognition methods such as Partial Least Squares-Discriminant Analysis (PLS-DA) and Soft Independent Modelling of Class Analogies (SIMCA), an approach that was also later exploited by Scappaticci et al. [23]. With this approach, the authors were able to discriminate between almonds cultivated in the Avola area from almonds coming from other Italian territories. More recently, Salvo et al. [24] used high-resolution magic angle spinning nuclear magnetic resonance (HR-MAS-NMR) to statistically distinguish the Avola almonds from other marketed products, based on the NMR profile that accurately reflected the higher relative content of sucrose with respect to fatty esters. Additionally, methods based on DNA analysis recently developed by Di Guardo et al. [25,26] could allow researchers to verify the genetic fingerprint of the cultivar.

Thanks to the collaboration with the *Consorzio di tutela della Mandorla di Avola*, a project has been started aiming at favoring the achievement of the IGP (Indicazione Geografica Protetta—Protected Geographical Indication) brand for this product, in order to protect the local production.

In this work, we used microelements determined by means of inductively coupled plasma—optical emission spectroscopy (ICP-OES) and inductively coupled plasma—mass spectrometry (ICP-MS) as chemical descriptors for (1) tracing the almond production chain, from soil to almond, (2) distinguishing samples of *Mandorla di Avola* almonds from almonds coming from California and Spain, which are usually employed as substitutes in pastry, and from other Italian provenances. ICP techniques are recognized as among the best techniques to determine the elemental contents of foods [27]; in particular, ICP-MS has the suitable sensitivity to reach the low or very low concentration level of many elements in foods, so that many chemical descriptors could be determined. This feature has been exploited in several studies of food authentication [28,29,30].

## 2. Materials and Methods

### 2.1. Reagents

To prepare sample and standard solutions, high-purity water (HPW) was generated with a Milli-Q (Milford, MA, USA) apparatus. The resulting resistance was 18 MΩ·cm.

Acids (nitric acid 69% and hydrochloric acid 37%) and hydrogen peroxide 30%, all of TraceSelect purity grade, were purchased from Fluka (Milan, Italy).

Polypropylene vials were used for sample and standard solution storage. Polystyrene vials were used for ICP analysis in an autosampler system. All the plastic vials were kept in 1% nitric acid and then rinsed with high-purity water when necessary. Multi-element stock solutions, purchased from Inorganic Ventures (Lakewood, NJ, USA), were used for external calibration and internal standardization.

### 2.2. Sample Collection

Samples of soil, shelled almonds, and almond tree leaves were provided by suppliers located in the Communes of Avola, Noto, and Pachino (province of Siracusa). In particular, samples of soils from almond groves were withdrawn at a depth of 30 cm in order to exclude external contamination. Two samples of almond pastes were also provided, one from the Avola area and one from Sicily but outside the Avola area.

### 2.3. Sample Treatment

#### 2.3.1. Soil

The pretreatment of soil included drying the samples at 120 °C overnight, sieving at φ 0.2 mm, and acid extraction aided by microwaves. For this task, aliquots of ca. 0.5 g were put in PTFE vessels and subjected to extraction with 2 mL of hydrogen peroxide 30% and 6 mL of aqua regia using a Start D microwave oven system (Milestone, Sorisole, Italy). Care was taken to add hydrogen peroxide before aqua regia (not the contrary), to limit the violent development of carbon dioxide caused by acid addition to calcareous soils. The temperature was then raised from 25 °C to 180 °C over 15 min and kept constant for 10 min, after which cooling was applied. After collecting the partially undissolved resulting mixture, this was taken to 50 mL with HPW in a polypropylene tube and subjected to centrifugation at 6000 rpm for 10 min, after which the supernatant solution was collected. Solutions were diluted 1:100 with HPW prior to ICP analysis.

#### 2.3.2. Almonds and Other Vegetal Matrices

Almonds of Avola are made of mostly unsaturated fats (>50%), proteins (15–18%), carbohydrates (10%), and fibers (13%). The water amount is low (4–5%). After taking almonds off their husks, they were ground in a crucible, then dried in an oven at 80 °C overnight. Two different pre-treatment methods were used: acid digestion and dry ashing, both aided by microwaves:

- Acid digestion was applied to 0.5 g of almonds in PTFE vessels, then 1 mL of 30% hydrogen peroxide, 3 mL of nitric acid 69%, and 4 mL of HPW were added, and the vessels were put inside the microwave oven system. During the heating treatment, the temperature was increased from 25 °C to 180 °C over a 10 min period and the temperature was kept constant for 15 min. The resulting solution was taken to 50 mL with HPW in a polypropylene tube. Solutions were then diluted 1:10 with HPW prior to ICP analysis.
- Dry ashing was applied by weighting ca. 10 g of almonds into a porcelain capsule and put inside a Milestone (Sorisole, Italy) Pyro 260 microwave ashing system. The heating cycle was as follows: room temperature to 150 °C in 10 min; hold at 150 °C for 20 min; raise temperature up to 500 °C in 20 min; hold at 500 °C for 30 min; raise temperature up to 750 °C in 10 min; hold at 750 °C for 30 min; raise temperature up to 1000 °C in 10 min; hold at 1000 °C for 30 min. The different heating steps were chosen in order to remove specific groups of molecules. The resulting ash was completely dissolved in 2.0 mL of ultrapure concentrated nitric acid and taken up to 50 mL with HPW in a polypropylene tube. Solutions were then diluted 1:10 with HPW prior to ICP analysis.

The results of dry ashing were found to be preferable because all the analytes were above the detection limits, in particular the lanthanides which are relevant markers for traceability. Acid digestion, on the contrary, did not guarantee results above the detection limits for all lanthanides. For the specific detection of volatile elements (e.g., As, Cd, Hg, Pb), however, acid digestion was used in parallel.

The same combined procedure was applied to almond paste, almond husks, and almond tree leaves. The resulting solutions were diluted 1:10 with HPW prior to ICP analysis.

### 2.4. ICP-MS Analysis

For the elemental analysis at the trace and ultra-trace level, a Thermo Scientific (Waltham, MA, USA) iCAP^TM^ RQ inductively coupled plasma mass spectrometer with single quadrupole technology was used. The system includes an ESI (Omaha, NE, USA) PFA 100 MicroFlow nebulizer, a Peltier-cooled quartz spray chamber that operates at 3 °C, a 2.0 mm ID quartz injector, and a demountable quartz torch. Uptake from the samples was managed by means of an ESI (Omaha, NE, USA) SC-4 DX autosampler system. In order to overcome possible spectral interferences given by polyatomic ions, all the measurements were carried out in a configuration involving the Collision Cell Technology (CCT) used with He gas at 3.5 mL/min and a kinetic energy discrimination (KED) barrier of 2 V; in this way, it was possible to minimize the interferences given by oxides in the determination of lanthanides (e.g., ^141^Pr^16^O on ^157^Gd). Despite the use of the CCT-KED device, the performances were comparable between standard and KED mode in terms of sensitivity (Ce > 500 k cps/ppb in both modes), thanks to the efficiency of Qcell flatpole. The entire system was managed by a PC via the Qtegra^TM^ v. 2.10.4345.136 software. The instrumental parameters used are listed in Table 1. The total acquisition time was 180 s for three replicates.

The precision, calculated on a stability test performed by monitoring ^7^Li, ^59^Co, ^115^In, ^140^Ce, and ^238^U, was better than 2%. The instrumental precision, calculated on the three replicates carried out on each sample, was better than 2% for trace and ultra-trace elements. The overall precision of the method, involving both sample preparation and instrumental analysis, calculated on five genuine replicates, was better than 5%.

Standard solutions were prepared in 1% nitric acid at 100, 10, 1, and 0.1 µg/L level, starting from CCS-1 (100 ppm Rare Earth, U, Th ICP Standard in 7% *v*/*v* Nitric Acid), CCS-2 (100 ppm Precious Metals ICP Standard in 10% *v*/*v* Hydrochloric Acid), CCS-4 (100 ppm Alkali, Alkaline Earth, Non-Transition Elements ICP Standard in 7% *v*/*v* Nitric Acid), CCS-5 (100 ppm Fluoride Stable Elements ICP Standard in 7% *v*/*v* Nitric Acid/1.0–1.9% *v*/*v* Hydrofluoric Acid), and CCS-6 (100 ppm Transition Elements ICP Standard in 7% *v*/*v* Nitric Acid) Inorganic Ventures (Christiansburg, VA, USA) multi-element standard solutions.

Limits of detection (LOD) and limits of quantification (LOQ), calculated as 3 and 10 times the standard deviation of blank measurements, respectively, can be found in another publication of the authors [31].

### 2.5. ICP-OES Analysis

For the elemental analysis of major and minor elements, a Spectro (SPECTRO Analytical Instruments GmbH, Kleve, Germany) Genesis ICP-OES simultaneous spectrometer was used. The instrumental parameters used are listed in Table 2. The total acquisition time was 140 s for three replicates.

Standard solutions were prepared in 1% nitric acid at 10, 5, 1, 0.5, and 0.1 mg/L starting from CCS-4 and CCS-5 Inorganic Ventures (Christiansburg, VA, USA) multi-element standard solutions.

Limits of detection (LOD) and limits of quantification (LOQ), calculated as 3 and 10 times the standard deviation of blank measurements, respectively, can be found in another publication of the authors [31].

### 2.6. Analysis of Certified Samples

The reliability of the analytical methods used was tested by analyzing two certified standard materials. For soil, we used SRM 2586 (*Trace Elements in Soil Containing Lead from Paint*) certified material from NIST. For vegetal matrices, i.e., almond tree leaves, almonds, almond husks, and almond paste samples, we chose SRM-1573A (*Tomato leaves*) from NIST, a material with a prevailing organic matrix. The results can be found in a very recent publication [32] and showed an acceptable agreement between certified and observed concentration values.

### 2.7. Data Analysis

For classification of samples, multivariate pattern recognition methods were used. Classification was carried out using XLSTAT (Addinsoft^TM^, Paris, France) v. 2012.2.02 software, running as an add-on for Microsoft Excel 2010 (Microsoft Corporation, Redmond, WA, USA).

## 3. Results

The production chain of almonds studied in this work was composed of the following stages:

- the soil on which the almond trees grow;
- the leaves of the almond tree;
- almonds;
- almond husks.

Four complete chains were analyzed.

### 3.1. Traceability of the Almond Chain

The concentration values determined by ICP-MS (trace elements) and ICP-OES (major and minor elements) were normalized with respect to Ce, which is always performed in similar works. Data were normalized in each sample by dividing the concentration of all elements for the Ce concentration in that sample. The reason for this normalization is to allow a better comparison among the different stages, considering that soils have concentrations from two to four times higher than the other matrices. The idea is to verify what elements behave like cerium along the food chain, with a focus on the other lanthanides.

**Figure 1:**
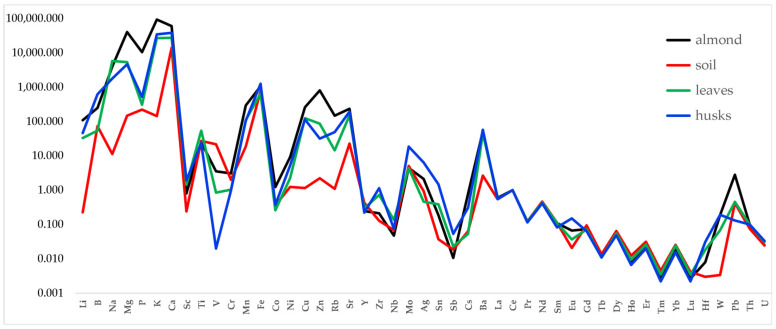
Distribution of elements in almond, soil, almond tree leaves, and almond husks. Data were normalized to Ce.

Figure 1 shows the Ce-normalized distribution of all the elements determined in an almond chain from Avola.

**Figure 2:**
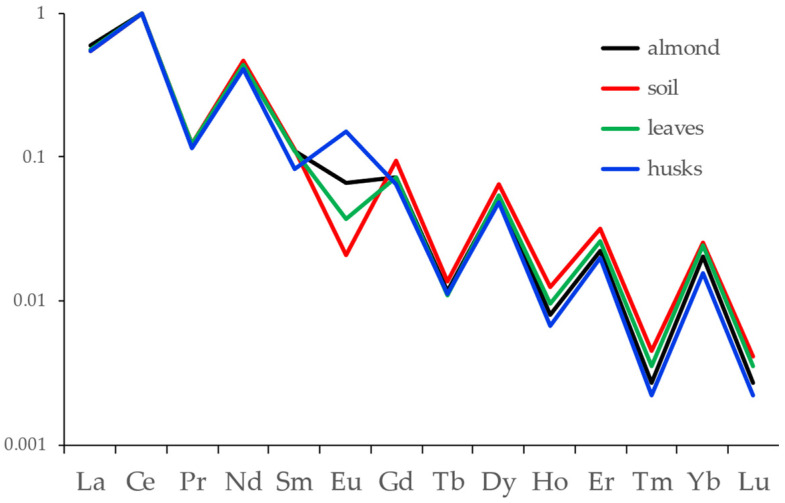
Distribution of lanthanides in almond, soil, almond tree leaves, and almond husks. Data were normalized to Ce.

As it has been observed in other works, the behavior of the lanthanides is quite different from that of the other elements. Many among the non-lanthanide elements appear to be fractionated with respect to the distribution in soil, as expected considering that some are nutrients for the almond tree (e.g., Mg, P, K), while some others are undesirable, if not toxic (e.g., V). This difference can be better appreciated from the graph reported in Figure 2, in which the lanthanides group only is shown.

The distribution of lanthanides in soil appears almost unaltered when passing from soil to leaves, as expected, and with minor changes from soil to almonds and almond husks. The slight fractionation that can be seen on the heavy lanthanides could be due to the higher uncertainty on the ICP-MS measurements, considering that these data correspond to concentrations close to the detection limits.

All the other three almond chains showed the same behavior, with a slight fractionation on the heavy lanthanides.

It must be noted that the results for Eu do not reflect its chemical or biological behavior along the chain: in fact, the use of a low resolution mass spectrometer for ICP-MS analysis did not allow us to avoid the positive interference of ^16^O^135^Ba^+^ and ^16^O^137^Ba^+^ ions on, ^151^Eu and ^153^Eu, respectively. Considering that Ba is from 100 to 10.000 times higher than Eu in the different stages and that the instrumental conditions used in the ICP-MS analysis allowed us to keep the oxide formation at ca. 0.5% of the element, these results indeed reflect the behavior of Ba along the chain (since Ba^2+^ is an ion vicariant of Ca^2+^, it is actively absorbed in leaves, almonds, and almond husks), rather than that of Eu.

### 3.2. Authentication on the Avola Almond

The element distribution was used to verify whether it was possible to authenticate the *Mandorla di Avola* variety with respect to almond varieties cultivated in other countries. The importance of this authentication scheme is because in many instances, sweets and other preparations claiming to contain the *Mandorla di Avola* variety do not contain it at all; rather, they are prepared with almonds from foreign provenances.

To set up the authentication scheme, a dataset of 47 elements was used. The samples were as follows:

- eight samples of *Mandorla di Avola* variety;
- six samples from California;
- two samples from Spain;
- six samples from Sicily;
- two samples from Italy (unspecified region);
- two samples of almond paste, one made from Avola almonds and one from foreign almonds.

The dataset was therefore composed of 26 samples × 47 variables. The samples were divided in two groups: nine samples of the *Mandorla di Avola* variety (eight almonds and one paste), and seventeen “foreign” samples (sixteen almonds and one paste), including almonds from Sicily but of a different variety and provenance.

**Figure 3:**
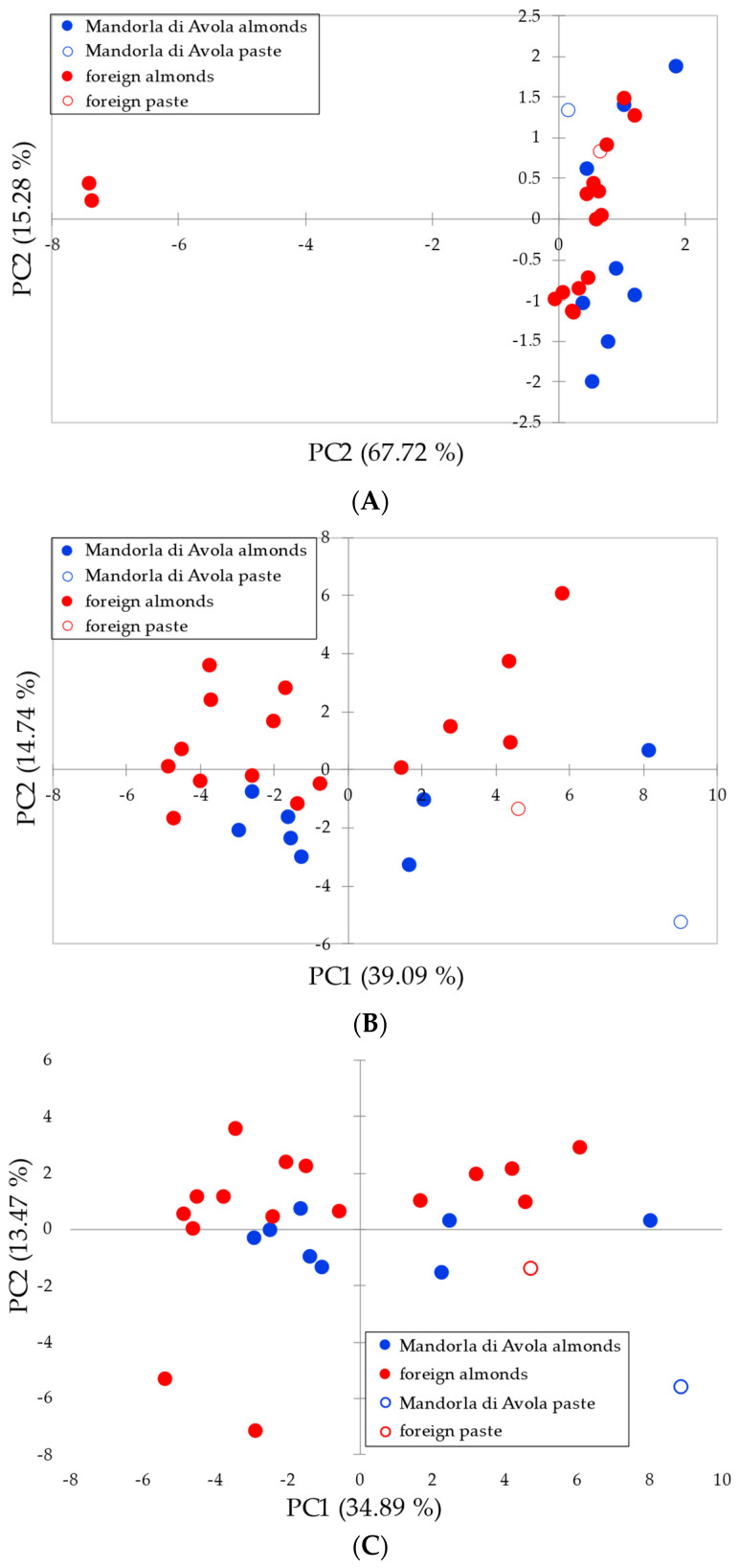
PC1 vs. PC2 plot of the data from ICP analysis of almonds: (**A**) using ICP-OES variables, (**B**) using ICP-MS variables, and (**C**) using ICP-OES + ICP-MS variables.

Data were transformed according to autoscaling, so that z-scores were used for the multivariate analysis. Principal Component Analysis (PCA) was firstly attempted to see whether the samples were clustered according to the geographic provenance. Two different models were calculated, one using as variables only the elements determined by means of ICP-OES, and the other using as variables only the elements determined by means of ICP-MS. The results of the PCA are shown in Figure 3A,B, respectively.

As it can be seen, the results are different: while the set of ICP-OES variables gave an unsatisfactory discrimination, the set of ICP-MS variables allowed a fairly good separation. The samples of the *Mandorla di Avola* (thereafter MdA in the figures) almonds mostly cluster at negative values of PC2; the same holds true for the sample of almond paste (white circle with blue outline). Foreign samples mostly cluster at positive values of PC2, but some samples and the almond paste (white circle with red outline) are far from this group. The overall separation was far from ideal. A similar result was obtained by using all the variables available (Figure 3C).

To improve the classification, a one-way ANOVA test was used to identify the variables that better allow a discrimination between *Mandorla di Avola* samples and the foreign samples. Mn, P, Pb, and Fe resulted in higher scores (Table 3) and were successively selected as descriptors.

Despite the fact that lanthanides were demonstrated to be good markers for traceability, as shown before, in this particular scheme of classification they were not efficient. In fact, their F-values were all lower than 1. This behavior is unusual if compared to similar studies on other vegetable food chains [32,33] and will be elaborated on later.

**Figure 4:**
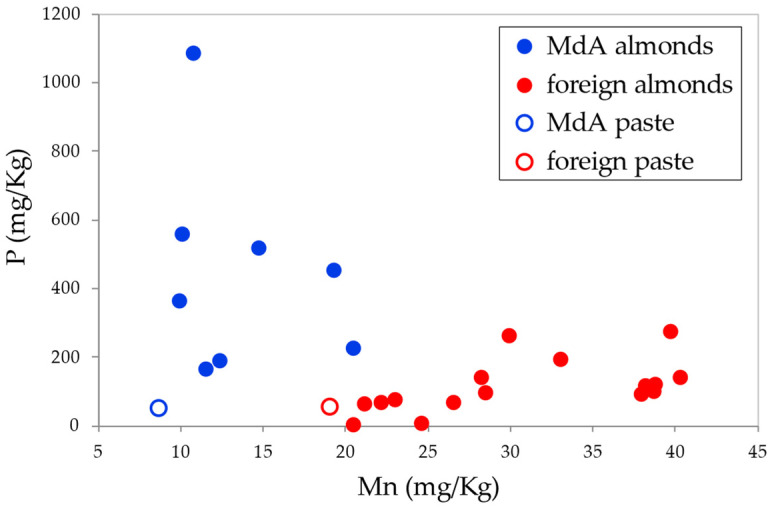
Biplot Mn vs. P.

The most discriminating variables resulted to be Mn and P. A simple biplot of Mn vs. P (Figure 4) allowed us to obtain a good discrimination among the two groups.

Foreign samples are characterized by a higher Mn content and a lower P content. The samples of almond paste produced with *Mandorla di Avola* almonds and with foreign almonds (white circle with blue and red outlines respectively) correctly clustered close to the respective groups.

**Figure 5:**
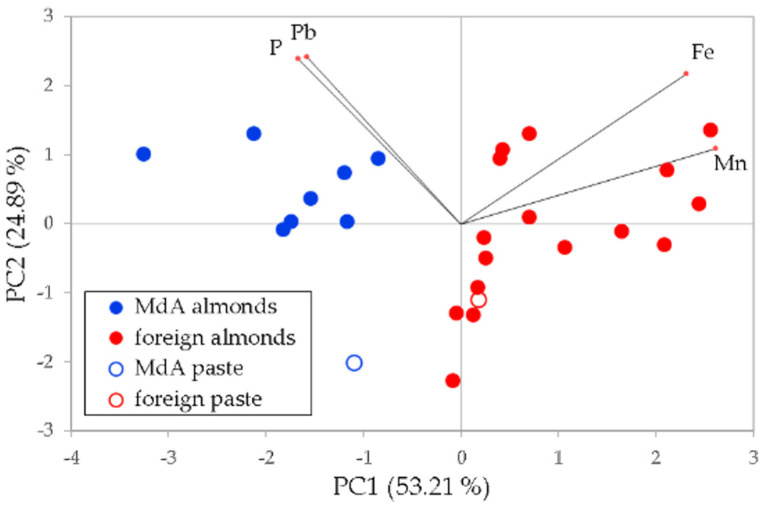
PC1 vs. PC2 plot using Fe, Mn, P and Pb as variables (black lines point to the loadings of the original variables).

The classification was improved by carrying out a PCA after selecting the four most discriminating variables according to the ANOVA test, that is, Mn, P, Pb, and Fe. As it can be appreciated in the PC1 vs. PC2 biplot reported in Figure 5, accounting for 78.10% of total variance, the *Mandorla di Avola* samples cluster at negative values of PC1, being richer in P and Pb, while all samples from other countries, richer in Fe and Mn, cluster at positive values of PC1.

The loadings coefficients of the four variables on PC1 and PC2 are shown in Table 4.

Again, the samples of almond paste clustered well with their respective groups, with the one produced with *Mandorla di Avola* almonds (white circle with blue outline) being on the negative side of PC1, and the one produced with foreign almonds (white circle with red outline) centered inside its group. The loading coefficients explain this scheme well: in fact, Mn and Fe have very high positive values on PC1, according to the higher concentrations of these elements in foreign almonds, while P and Pb both have negative values on PC1, according to the higher concentrations of these elements in *Mandorla di Avola* almonds. No particular behavior was noted on PC2.

## 4. Discussion

### 4.1. Traceability of the Almond Chain

In this study, the distribution of lanthanides appeared to be maintained fairly constantly when passing from soil to almond, so that it can be considered a fingerprint of that soil. Previous studies on other food chains highlighted two kinds of behaviors, as far as the lanthanides distribution is concerned: in the case of olive oil [33], a very good matching between the fingerprint of soil and food was found, while in the case of milk [34] and honey [32], some fractionation occurred, in particular on the heavy lanthanides; it was hypothesized that the second behavior was due to the animal metabolism of cows and bees, respectively. In the chain of wine [35], on the contrary, significant fractionation occurred, most probably due to the working stages after the fermentation of must. From the results displayed in Figure 2, we can state that in the case of almonds, the chain can be well traced from soil to fruit.

### 4.2. Authentication on the Avola Almond

Microelements have been frequently used to determine the provenance of foods. The topic has been thoroughly discussed in recent reviews [28,36]. Apart from lanthanides, many other elements at trace- or ultra-trace levels were found to be useful for this task. However, every classification scheme utilizes specific chemical markers. In the present study, the role of lanthanides appeared to be negligible for the discrimination of the *Mandorla of Avola* almonds from almonds of foreign provenances. Nevertheless, it was possible to find other chemical descriptors, i.e., Mn, P, Pb, and Fe, which allowed us to obtain a good classification scheme. The reasons why these specific elements were so efficient in the classification is not at present known; further studies must be carried out to clarify their role in the relationship between soil and almonds.

It must be considered that the concentration of some microelements, in particular Mn and P, could be influenced by the agricultural practices, since they are contained in fertilizers. However, the agricultural practices used in the areas where the *Mandorla di Avola* variety is grown are particularly eco-compatible and strictly controlled (e.g., treatments with chemical products are limited if not excluded). In addition, the overall area has a dominant vocation for agriculture, and this strongly limits the possibility of soil contamination from industrial activities. In the end, we can hypothesize that the contributions from anthropical sources in the elemental composition of the soil where the *Mandorla di Avola* variety is grown are not relevant.

Among the four chemical descriptors useful for the discrimination, Mn, Pb, and Fe were determined by means of ICP-MS, while P was determined by means of ICP-OES. This involves the need to have both techniques at one’s disposal, which can be uncommon for several laboratories. However, the determination of P can be achieved with ICP-MS also, using the ^31^P isotope and a proper dilution of the samples, so as to limit the analytical needs of the proposed method to a single technique.

## 5. Conclusions

The determination of microelements by means of ICP techniques has demonstrated its utility to certify the quality of the *Mandorla di Avola* almonds. These chemical descriptors can be exploited both for the traceability and authentication of this valuable product. In the first case, the comparison of the distribution of lanthanides suggests that the original fingerprint of soil is transmitted to leaves and almonds, allowing one to establish a connection between soil and almonds. In the second case, it was possible to individuate some chemical descriptors (Mn and P being the best ones) to develop a classification scheme for the discrimination of the *Mandorla di Avola* almonds from almonds of other geographic provenances. It must be noted that, due to the small number of samples, these must be considered as preliminary results. A more extensive study with more samples will follow.

## Figures and Tables

**Table 1:** Instrumental parameters for ICP-MS analysis.

Parameters	Value
Forward power	1550 W
Plasma gas flow	14.0 L/min
Nebulizer gas flow	0.9 L/min
Auxiliary gas flow	0.8 L/min
Isotopes used	^45^Sc, ^49^Ti, ^51^V, ^52^Cr, ^55^Mn, ^57^Fe, ^59^Co, ^60^Ni, ^63^Cu, ^66^Zn, ^85^Rb, ^88^Sr, ^89^Y, ^90^Zr, ^93^Nb, ^95^Mo, ^107^Ag, ^118^Sb, ^121^Sb, ^133^Cs, ^137^Ba, ^139^La, ^140^Ce, ^141^Pr, ^146^Nd, ^147^Sm, ^153^Eu, ^157^Gd, ^159^Tb, ^163^Dy, ^165^Ho, ^166^Er, ^169^Tm, ^172^Yb, ^175^Lu, ^179^Hf, ^182^W, ^208^Pb, ^232^Th and ^238^U
Internal standards	^103^Rh, ^115^In and ^193^Ir at 10 μg/L
CeO^+^/Ce^+^	<0.5% in KED mode

**Table 2:** Instrumental parameters for ICP-OES analysis.

Parameters	Value
Plasma observation	axial
Forward power	1400 W
Plasma gas flow	12.0 L/min
Nebulizer gas flow	0.96 L/min
Auxiliary gas flow	0.90 L/min
Wavelengths used	B (249.773 nm) Ca (317.993 nm) K (766.491 nm) Li (670.780 nm) Mg (285.213 nm) Na (589.592 nm) P (177.495 nm)
RF generator	40 MHz
RF power	1300 W

**Table 3:** F values for the most discriminating elements.

Elements	F Value
Mn	36.72
P	17.91
Pb	12.14
Fe	5.98

**Table 4:** Loadings coefficients for the 4 variables on PC1 and PC2.

Variables	PC1	PC2
Fe	0.806	0.518
Mn	0.911	0.260
P	−0.586	0.571
Pb	−0.552	0.579

## Data Availability

The original contributions presented in the study are included in the article; further inquiries can be directed to the corresponding author.
